# Interspecies Transmission of Animal Rotaviruses to Humans: Reassortment-Driven Adaptation

**DOI:** 10.3390/pathogens14121230

**Published:** 2025-12-02

**Authors:** Toyoko Nakagomi, Osamu Nakagomi

**Affiliations:** Department of Hygiene and Molecular Epidemiology, Graduate School of Biomedical Sciences, Nagasaki University, Sakamoto, Nagasaki 852-8523, Japan; onakagom@nagasaki-u.ac.jp

**Keywords:** rotavirus, genetic reassortment, interspecies transmission, whole-genome sequencing, genotype constellation, genogroup

## Abstract

*Rotavirus alphagastroenteritidis* (rotavirus) infects a broad range of hosts, including humans and various animal species. Its genome comprises 11 segments of double-stranded RNA, making it highly prone to genetic diversity through gene reassortment. Although rotavirus strains are typically host-specific, novel human strains with global impact often originate from interspecies transmission of animal rotaviruses. This review explores the critical role of interspecies transmission coupled with genetic reassortment in rotavirus adaptation to humans, contextualizing key studies and methodological advances. Central to this progress was the development of tools to analyse entire genomes and distinguish homologous from heterologous strains. We trace the evolution from RNA-RNA hybridisation to whole-genome sequencing, which underpins genotype constellation and sub-genotype phylogeny. A decade-long surveillance of the bovine-like G8 rotavirus in Vietnam offers a compelling model: for an animal rotavirus to become a successful human pathogen, it must replace its animal-derived genes with human-derived counterparts through reassortment. Retaining the animal-origin G8 VP7 gene is enabled by acquiring a compatible human VP4 gene (specifically P[8]) and DS-1-like backbone genes. Building on this model of reassortment-driven adaptation, our investigation into the unusual G1P[6] strain AU19, of wholly porcine origin, supports the hypothesis that the predominant human G1 rotavirus also evolved from a successful interspecies transmission event. Phylogenetic analysis suggests the ancestral human G1 gene emerged from a porcine rotavirus between 1915 and 1948, later reassorting with human strains to acquire Wa-like backbone genes, ultimately becoming a stable and dominant part of the human rotavirus population. In conclusion, genetic reassortment is a key mechanism transforming sporadic zoonotic events into sustained human-pathogens, although other factors remain to be fully defined. We conclude by highlighting key areas for further research.

## 1. Introduction

**Figure 1:**
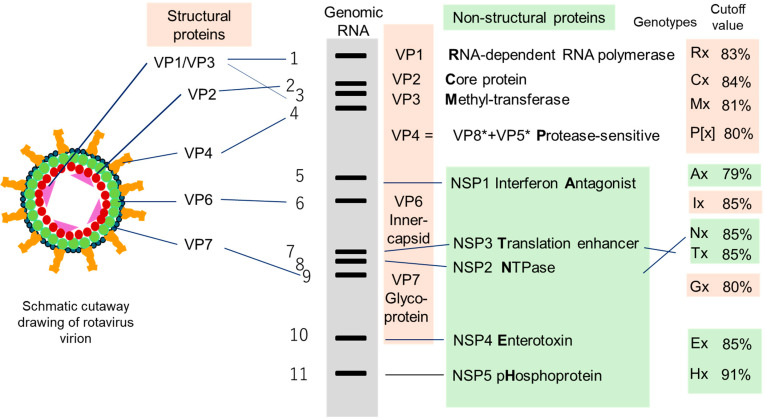
Schematic Structure of the Rotavirus Virion and Genome. The diagram shows the virion in cross-section (triple-layered particle) and its 11 double-stranded RNA (dsRNA) segments separated on a gel. Structural Layers: The innermost layer is composed of VP2 (magenta), the middle layer of VP6 (green), and the outer surface of VP7 (blue) with projecting VP4 spikes (orange). An asterisk attached to VP5* and VP8* indicates that they are modified post-translationally. Genomic RNA: Segments are numbered 1 (slowest-migrating band) to 11 (fastest). All segments are monocistronic, except for segment 11, which is bicistronic and encodes non-structural proteins NSP5 and NSP6. Nucleotide identity cutoff values used for genotype assignment throughout this review adhere to the standards recommended by the Rotavirus Classification Working Group (RCWG). Specifically, the cutoff values for the VP1, VP2, VP3, VP4, VP6, VP7, NSP1, NSP2, NSP3, NSP4 and NSP5/6 genes are 83%, 84%, 81%, 80%, 79%, 85%, 85%, 85%, 80%, 85%, and 91%, respectively. Created anew for this article.

The species *Rotavirus alphagastroenteritidis*, more commonly called Rotavirus A, is a member of the genus *Rotavirus* within the family *Sedoreoviridae* [1,2] and will hereafter be referred to simply as rotavirus for convenience. The genome of rotavirus consists of 11 strands of double-stranded RNA, each of which encodes a single viral structural protein (VP1-VP4, VP6 and VP7) or non-structural protein (NSP1-NSP4), except for genome segment 11, which codes for NSP5/6. The RNA genome is encased in a virion with icosahedral symmetry that comprises three-layered capsid proteins (Figure 1) [3]. Rotavirus infects a wide range of hosts, including humans and various animal species. While each host is typically infected by its own host-specific (homologous) strains, rotaviruses originating from different animal hosts (heterologous strains) can occasionally infect humans and cause disease. This phenomenon underpins the concept of interspecies transmission of animal rotaviruses to humans [4,5,6,7,8]. In this review, the term animal rotaviruses is used to denote non-human animal rotaviruses.

This review aims to explore and discuss how the concept of interspecies transmission has evolved over the years through methodological advancements that enable the distinction between homologous and heterologous rotavirus strains by examining the entire genome. To achieve this aim, exemplary studies were selected to contextualize their findings within the framework of the current understanding of rotavirus interspecies transmission. Consequently, the selection of papers is not exhaustive and reflects a degree of bias toward studies in which we were directly involved, allowing for re-examination of original data and unpublished background information to provide deeper interpretation.

To begin, consider the following illustrative case. During the summer of 1995, a baby boy from Jerusalem—an urban area with no cattle—was visiting relatives in Petah Tikva, a city near Tel Aviv that housed several cow sheds and petting farms for children. During the visit, his mother took him to one such farm to see cows and calves for the first time. A few days later, the child developed diarrhoea and was examined by a paediatrician. Laboratory analysis of his faecal sample confirmed rotavirus infection. From this clinical history, the infection was suspected to be caused by a bovine rotavirus from the beginning. But it was 18 years later that a thorough investigation by whole-genome sequencing concluded that the child was infected with a G6P[1] bovine rotavirus [9].

While this case represents a clearly documented spillover event, such direct evidence of animal contact preceding illness is exceptionally rare. In most reports based on surveillance data, the zoonotic link is inferred rather than confirmed, typically referencing generalised ecological conditions—such as human proximity to domestic or wild animals in rural regions of Asia and Africa—rather than direct, observed contact.

## 2. Two Key Questions Regarding Interspecies Rotavirus Transmission

The concept of interspecies transmission is straightforward, but exploring it in real-world samples requires answering two key questions. First, how can one find an animal rotavirus strain among the sheer number of human strains? It is like finding a needle in a haystack, and one needs some tool to find it like a magnet. While significant distinction lies in the minimal infectious dose, which is tens of thousands of times smaller for homologous hosts than for heterologous ones, as demonstrated in a mouse model [10,11,12], applying this criterion to clinical specimens is impractical. One realistic approach applicable to clinical samples is to leverage the lower degree of genomic RNA homology between rotavirus strains from different host species than within the same host species. It was hypothesized that genetic homology would be substantially higher among homologous strains than between heterologous ones, making such distinctions critical. However, even at the end of the twentieth century, sequencing the whole genome of a field isolate remained technically challenging. Thus, it was necessary to develop nucleic acid hybridisation assays that can demonstrate the presence of a heterologous rotavirus strain of animal origin.

The second question is the extent to which interspecies transmission contributes to rotavirus evolution. If interspecies transmission is limited to the individual patient, lacking any long-term, inheritable consequences, it does not impact evolution. Here, evolution refers to the creation of genetic diversity, a rich source of variants on which selection pressures act (Mayr, 1994) [13]. Thus, the second question relates to whether interspecies transmission can serve as a driving force in shaping the genetic diversity of the rotavirus population.

## 3. What Is a Strain of Rotavirus and How to Define It?

The term “strain” of rotavirus is inherently ambiguous and has been used variably across the research community. Prior to the metagenomic era, rotavirus strain was referred to as a plaque-purified clone isolated in cell culture from a given patient. Then, the strain, often possessing distinct biological and genetic properties, was exchanged among researchers as a common practice.

**Figure 2:**
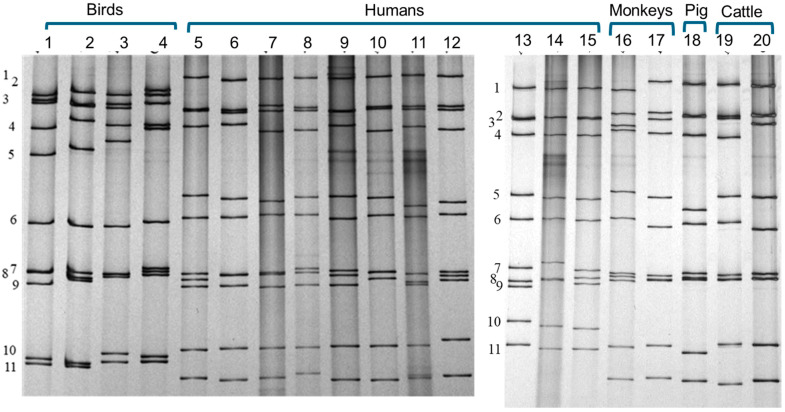
Polyacrylamide gel electrophoresis (PAGE) of the genomic RNAs from Avian and Mammalian Rotavirus Strains. The unique migration profile (or band pattern) for each strain is termed an electropherotype, which serves as a molecular fingerprint for distinguishing and characterising different rotavirus strains. This figure is a composite image assembled from two polyacrylamide gels to visually demonstrate the characteristic differences in electropherotypes between avian rotavirus strains (lanes 1–4) and mammalian rotavirus strains (lanes 5–20). Note that in mammalian strains (lanes 5–20), the eleven genome segments migrate into four distinct groups (segments 1–4, segments 5 and 6, segments 7–9, and segments 10 and 11). This grouping is characteristically altered in avian rotaviruses (lanes 1–4): segment 5 migrates very slowly, separating it from segment 6, and segments 10 and 11 nearly co-migrate. Predicting the specific host species origin of a rotavirus based solely on its electropherotype is otherwise impossible. Statement of origin and novelty: This image does not contain new scientific data. The RNA samples were extracted from well-known strains maintained in the authors’ laboratory and analysed in-house. The specific electrophoretic image shown was generated and compiled in the authors’ laboratory using established strains, hence requires no external permission for reuse in this academic context. Methodological detail for transparency: Samples were loaded onto 0.75-mm-thick polyacrylamide gels (4% stacking, 10% separating) and run for 16 h at 8 mA per gel. Following electrophoresis, gels were silver-stained. Lanes: 1: pigeon rotavirus PO-13 G18P[17]; 2: turkey rotavirus Ty-1 G17P[17]; 3: turkey rotavirus Ty-3 G7P[17]; 4: chicken rotavirus Ch-1 G19P[17]; 5: human rotavirus Wa G1P[8]; 6: AU007 G1P[8]; 7: IGV80 G1P[8]; 8: AU801 G1P[8]; 9: RIX4414 G1P[8]; 10: MO G3P[8]; 11: M37G1 P[6]; 12: AU32 G9P[8]; 13: AU64 G1P[4]; 14: DS-1 G2P[4]; 15: KUN G2P[4]; 16: simian rotavirus SA11 G3P[2]; 17: rhesus monkey rotavirus RRV G3P[3]; 18: porcine rotavirus OSU G5P[7]; 19: bovine rotavirus NCDV G6P[1]; 20: UK G6P[5].

A rotavirus strain can be characterized by neutralisation specificity (serotype) but a serotype is dually defined by two genome segments, i.e., the VP7 and VP4 genes, independently from the other nine genome segments (Figure 1). From the early years of rotavirus research, a method to analyse the entire genome has been polyacrylamide gel electrophoresis of genomic RNA [14,15]. Polyacrylamide gel electrophoresis reveals the migration patterns of the virus’s 11 RNA segments, producing a unique profile known as an electropherotype (Figure 2). This technique offers a rapid and reliable means of distinguishing between strains: two distinguishable electropherotypes conclusively indicate that two samples contain genetically distinct rotavirus strains.

In the previously mentioned Israeli boy’s case, polyacrylamide gel electrophoresis revealed that the electropherotype of the rotavirus isolated from the boy differed from that of NCDV, a commonly used laboratory strain of bovine rotavirus. This distinction was crucial, as it effectively ruled out the concern of laboratory contamination at an early stage of the study.

Another interesting finding is the characteristic electropherotypes of avian rotaviruses, which are clearly distinguishable from those of mammalian rotaviruses (Figure 2). While this distinction is not definitive proof of avian origin, it provides strong supporting evidence that warrants further investigation.

The following is a good example: When over 1400 bovine faecal specimens collected in Germany were screened, a few rotavirus strains with unusual electropherotypes were detected by polyacrylamide gel electrophoresis. One such strain, designated 993/83, was successfully isolated in cell culture [16]. The distinctive electropherotype of 993/83 prompted an analysis by RNA-RNA hybridisation. This analysis, further supported by neutralization assays, concluded that strain 993/83 was of avian rotavirus origin, representing the first case of rotavirus transmission across classes in vertebrates [17]. Initially, there was scepticism about whether an avian rotavirus could infect mammals; however, it was unequivocally shown that mice were experimentally infected with a pigeon rotavirus, PO-13 [18,19].

While useful for defining rotavirus strains in molecular epidemiological studies, electropherotyping lacks the ability to reliably distinguish between homologous and heterologous strains, with the notable exception of the characteristic electropherotypes of avian rotaviruses (Figure 2). This discriminatory ability is crucial for investigating interspecies transmission.

## 4. Advancement of Analysing the Whole Genome: RNA–RNA Hybridisation

From the 1980s until the advent of whole-genome sequencing technologies in the early 2000s, the primary method for analysing the whole genome of rotavirus strains was RNA–RNA hybridisation in solution [20,21]. Under high-stringency conditions, theoretically, up to 18% nucleotide mismatches were allowed to form hybrid bands [22]. This technique enabled researchers to assess genomic RNA homology which was impossible by electropherotyping. Based on the pattern of hybrid formation, wild-type human rotavirus strains were classified into three distinct groups, which we termed “genogroups”; i.e., Wa, DS-1, and AU-1 [23,24,25].

**Figure 3:**
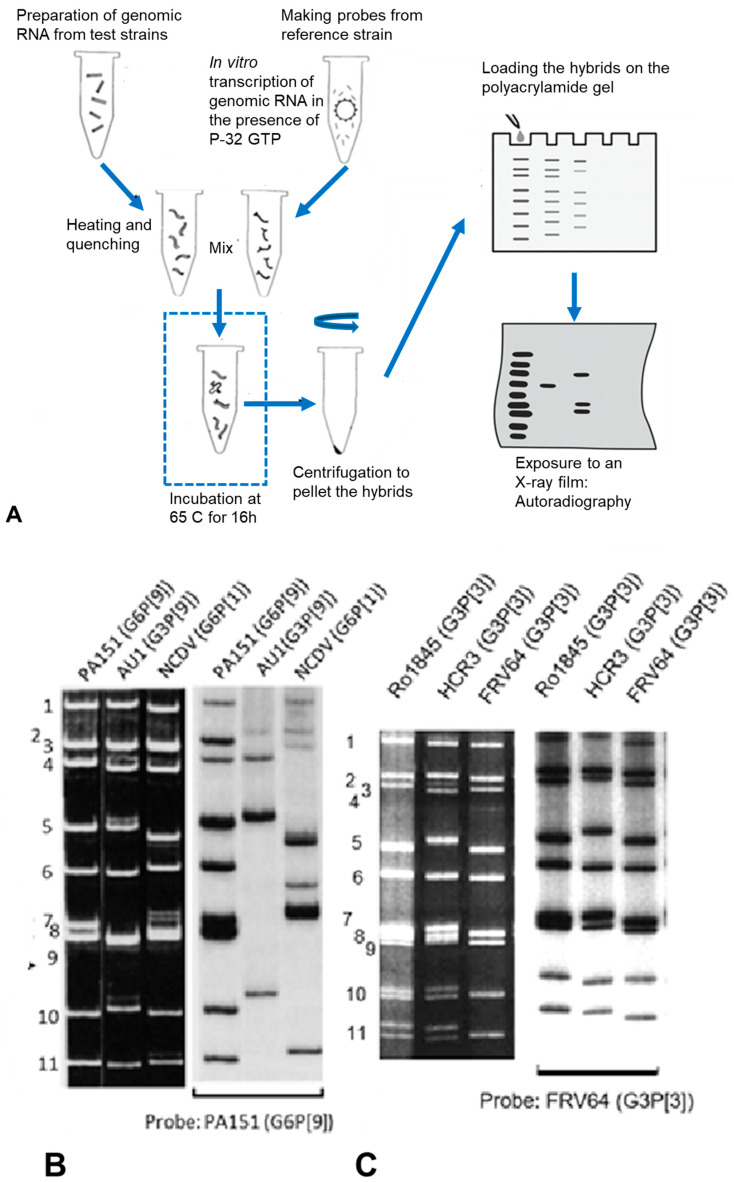
Schematic diagram illustrating the RNA-RNA hybridisation procedure to generate an autoradiogram. (**A**): Genomic double-stranded RNAs are purified from the test rotavirus strain and heat-denatured. Separately, P32-labeled, positive-sense RNA probes (11 single-stranded transcripts) are generated from the reference strain by in vitro transcription, using purified double-layered particles containing the active viral RNA polymerase. The denatured test RNA and the labelled probes are mixed in a small reaction volume and incubated under high-stringency conditions (65 C for 16 h) to allow for hybridisation. Un-hybridised RNA probes are degraded during this process. The resulting RNA-RNA hybrids are recovered by ethanol precipitation and separated using polyacrylamide gel electrophoresis (PAGE). Then, the gel is dried and exposed to an X-ray film to produce an autoradiogram. (**B**): The PA151 probe forms 11 co-migrating bands in the homologous reaction. With heterologous reactions, PA151 hybridises to specific segments of AU-1 and other segments of NCDV. This segmented hybridisation pattern suggests PA151 is a reassortant formed between AU-1-like and NCDV-like parental strains. Note that the aberrant migration of hybrids precludes identification of the genome segment origin for each band. (**C**): The FRV64 (feline) probe yields an almost indistinguishable 11-band profile when hybridised with the genomic RNA of the human strain Ro1845 and the canine strain HCR3. This near-identical pattern indicates an extremely high sequence identity across all segments, suggesting whole-virion transmission (feline or canine) to the human child. Panel (**A**) was created for this article. Panels (**B**,**C**) were adapted from references [28,33], respectively with the permission of respective publishers.

Figure 3 illustrates briefly how RNA–RNA hybridisation assay in solution is performed. In the interpretation of the assay results, it deserves mention that unlike northern blot hybridisation [26], the migration of hybrid molecules formed in solution with many sequence mismatches results in slower and fainter migration on the gel. In such instances, aberrant migration precludes identification of the segment origin for each band although the intensity and the degree of aberrant migration on the gel are suggestive of the relative heterogeneity between the probe and genomic RNA.

RNA–RNA hybridisation analysis to explore genetic relationships between different animal rotaviruses revealed that they largely form host-species specific genogroups [27]. This confirmed that rotaviruses tend to be genetically similar within a particular animal species whereas they differ substantially between heterologous strains. In light of this observation, RNA–RNA hybridisation analysis of interspecies transmission cases revealed two distinct hybrid patterns [4,5] (Figure 3). In the first pattern, the entire genome exhibits homology with that of animal rotaviruses, suggesting interspecies transmission as whole virions [23,28]. In the second pattern, which is more frequently encountered, only certain genome segments hybridise with one probe, while the remaining segments hybridise with a different probe, thereby indicating the occurrence of genetic reassortment [29,30,31,32,33,34].

## 5. Further Advancement by Whole Genome Sequencing and Phylogeny

### 5.1. Defining a Genotype and Genotype Constellation

A major limitation of RNA–RNA hybridisation techniques was their inability to estimate the timing of genetic reassortment events—whether they occurred at the time of spillover events or gradually evolved during sustained transmission within the new host (i.e., human) population.

The ambiguity surrounding the timing of gene reassortment events has been resolved through advances in molecular phylogenetics including the coalescent model [35,36]. Nucleotide sequence data provided by whole-genome sequencing of wild-type strains have enabled researchers to distinguish between interspecies transmission as a contemporaneous event and as part of a longer-term evolutionary trajectory.

In this genomic era, traditional genogroup classification by RNA–RNA hybridisation assays was superseded by genotype constellations defined by quantitative criteria [37] (Figure 1). As polymorphisms were identified across all 11 genome segments, including the two outer capsid protein-coding segments (VP7 and VP4), a standardized nomenclature was adopted to describe the full genotype constellation: Gx–P[x]–Ix–Rx–Cx–Mx–Ax–Nx–Tx–Ex–Hx, where each “x” denotes the genotype number assigned to a specific gene segment [37,38].

There are two major and one minor genotype constellations among human rotaviruses; they are the Wa, DS-1, and AU-1 genotype constellations comprising G1/G3/G4/G9-P[8]-I1-R1-C1-M1-A1-N1-T1-E1-H1, G2-P[4]-I2-R2-C2-M2-A2-N2-T2-E2-H2, and G3-P[9]-I3-R3-C3-M3-A3-N3-T3-E3-H3, respectively [37,39]. The authorisation and numbering of a new genotype is carried out by the Rotavirus Classification Working Group [38]. As of this writing, 42 G, 58 P, 32 I, 24 R, 24 C, 24 M, 39 A, 28 N, 28 T, 32 E and 28 H genotypes are registered.

The shift to quantitative, sequence-based genotype constellations provides invaluable insight [37]. This modern approach, which defines genotypes based on the predefined cutoff value of nucleotide sequence identity, reveals the intricate interrelationships and shared genotypes between rotaviruses from different host species. Specifically, the cutoff values for the VP1, VP2, VP3, VP4, VP6, VP7, NSP1, NSP2, NSP3, NSP4 and NSP5/6 genes are 83%, 84%, 81%, 80%, 79%, 85%, 85%, 85%, 80%, 85%, and 91%, respectively.

For instance, a reference bovine rotavirus strain, NCDV, has the genotype constellation G6-P[1]-I2-R2-C2-M2-A3-N2-T6-E2-H3. In comparison, the human DS-1 strain has a constellation of G2-P[4]-I2-R2-C2-M2-A2-N2-T2-E2-H2. A direct comparison shows that these two strains share the same genotypes for six of their genome segments: VP6 (I2), VP1 (R2), VP2 (C2), VP3 (M2), NSP2 (N2), and NSP4 (E2). Based on these and related findings, Matthijnssens et al. hypothesized that DS-1-like human rotaviruses and bovine rotaviruses share a common animal origin, while Wa-like human rotaviruses are more closely related to porcine rotaviruses [37].

### 5.2. Classification Within a Genotype: Sub-Genotype Phylogeny

Although rotaviruses may share the same genotype, detailed analysis reveals distinct lineages across different host species. This underscores the limitations of genotype-level classification and the need for lineage-level resolution. The sub-genotype phylogeny framework proposed by Agbemabiese et al. [40] offers a powerful tool for tracing the evolutionary history of the backbone genes (the genes encoding the internal capsid proteins and non-structural protein genes) of DS-1-like strains.

For example, as mentioned in the preceding section (Section 5.1), the bovine strain NCDV and the human strain DS-1 share identical genotype 2 genes for VP6 (I2), VP1 (R2), VP2 (C2), VP3 (M2), NSP2 (N2), and NSP4 (E2). This identity strongly suggests a common ancestor. However, applying the sub-genotype phylogeny framework to these six genes reveals that they belong to significantly different lineages for all six genes, contradicting the assumed recent common ancestry. Specifically, the lineages for NCDV are VP6 (I2.X), VP1 (R2.XII), VP2 (C2.X), VP3 (M2.X), NSP2 (N2.XIII), and NSP4 (E2.XXIV), whereas those of DS-1 are consistently VP6 (I2.I), VP1 (R2.I), VP2 (C2.I), VP3 (M2.I), NSP2 (N2.I), and NSP4 (E2.I). Furthermore, there is significant nucleotide divergence, ranging from 12.9% for NSP2 to 14.7% for VP1, between the two strains across these six genes.

The evidence indicates that while some backbone genes of the human DS-1 strain likely originated in cattle (or other artiodactyls), these DS-1 genes have since become evolved to adapt to humans (humanised).

Comparable lineage-based tools for Wa-like and AU-1-like backbone genes are essential to deepen our understanding of rotavirus evolution.

## 6. From Spillover Event to Emerging Epidemic

### 6.1. Need for Timely Surveillance

Many papers on interspecies transmission emphasise the importance of surveillance. However, before a major outbreak occurs, it is difficult to distinguish between a dead-end spillover event and the one resulting in an epidemic emergence. Securing samples soon after an animal rotavirus strain crosses the species boundary is therefore vital. This allows researchers to track the adaptive mutations that are essential for establishing sustained human-to-human transmission. However, this is easier said than done because there were only two emerging epidemics caused by animal-derived rotavirus strains in the last two decades: the bovine-like G8P[8] strains, which emerged in Southeast Asia around 2013 [41], and the equine-like G3P[8] strains, which emerged in Australia and Thailand in 2013 [42,43]. Nevertheless, maintaining a prepared surveillance infrastructure can increase the chances of early detection.

### 6.2. Bovine-like G8P[8] Strains That Emerged in VIETNAM in 2014

#### 6.2.1. Observations in Vietnam

A case in point is the strain surveillance in Vietnam which provided us with a unique opportunity of early detection [44]. Until late 2014, the distribution of rotavirus genotypes in Vietnam was unremarkable, with G1P[8] and G2P[4] accounting for over 90% and up to 10% of rotavirus-positive samples, respectively. However, in November 2014, suddenly an unusual G8P[8] strain was detected in the southern city of Nha Trang. The number of G8P[8] samples gradually increased, and by 2015, the strain had spread north to Hanoi [41]. At that point in time, we thought that we were witnessing the emergence and early spread of a novel G8P[8] rotavirus strain. We speculated that this was a bovine G8 strain that had jumped into humans, as sequencing of the VP7 gene revealed that its most recent common ancestor was shared with an Indian bovine G8 strain [41].

**Figure 4:**
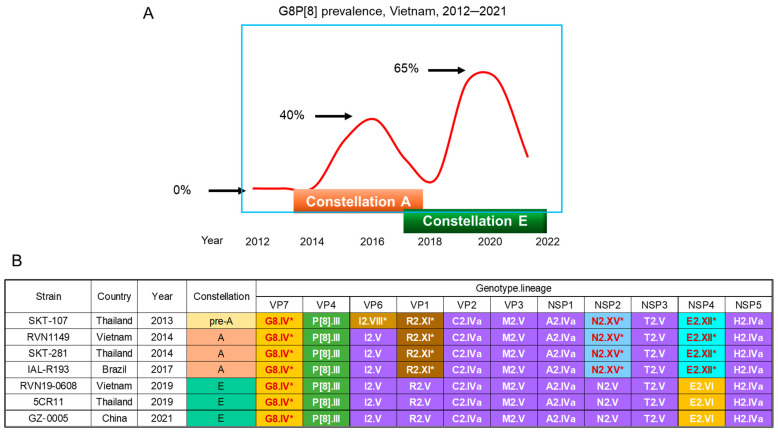
Prevalence and genotype/lineage constellation of bovine-like G8P[8] rotavirus strains. (**A**): Prevalence of bovine-like G8P[8] strains in Vietnam during the study period (2012–2021) is shown. Note that no G8P[8] strain was detected during the first two years of surveillance (2012–2013). The strain abruptly emerged in 2014 and peaked in 2016 and again in 2019–2020. Although all strains shared the DS-1-like genetic backbone, the lineage constellation of the dominantly circulating strain changed from Constellation A in the first wave to Constellation E in the second wave. (**B**): The genotype/lineage constellation of bovine-like G8P[8] strains representative of selected countries and years is displayed. A lineage marked with an asterisk (*) denotes that the lineage is likely derived from animal rotaviruses in the relatively recent past. (**A**). The figure was produced anew, based on our own data from ref. [44] which was published under the CC-BY license. (**B**). The table was produced anew, based on the data from ref. [44] and combined with additional information from refs. [45,46,47,48]. The lineage designation was according to ref. [40].

The outbreak occurred in two distinct waves, peaking in 2016 and again in 2019–2020 [44] (Figure 4). The strain eventually ceased to circulate in late 2021. Our decade-long monitoring of the G8 strain revealed a clear picture of the adaptive mutations that had likely driven the spread of the virus. Whole genome sequencing confirmed that all samples shared a common genotype constellation: G8-P[8]-I2-R2-C2-M2-A2-N2-T2-E2-H2; i.e., bovine-like G8P[8] with a DS-1-like genetic backbone. However, at a more granular lineage level, the strains were grouped into five genetic constellations two of which were dominant ones: constellation A and constellation E [44] (Figure 4). Interestingly, the most notable difference between the two constellations was the presumed origin of their backbone genes. The earlier strains, which belonged to constellation A, possessed bovine-derived genes in the VP1, NSP2, and NSP4 genes. In contrast, the later strains from constellation E had undergone a significant change: all three bovine-derived genes were replaced with their human counterparts through a process of genetic reassortment [44].

While our discussion is primarily focused on reassortment events involving VP1, NSP2, and NSP4, the successful spread needs to be understood as more granular molecular changes in these and other segments. As such, future research requires a comprehensive understanding of functional regions of each genome segment. To serve as a general resource for such studies (as discussed further in Section 11.1), we provide an overview of the known functions of each of the 11 rotavirus genome segments in Supplementary Table S1.

#### 6.2.2. Observations in Nearby Countries

Two relevant reports from the literature deserve mention to extrapolate our observations. First, an analysis of the seven Thai G8P[8] strains such as SKT-107 detected one year earlier than the earliest Vietnamese strain RVN1149 revealed that they had the same genome as constellation A, with one important exception: their VP6 gene belonged to lineage I2.VIII (Figure 4) [45]. This I2.VIII lineage was the one carried by a Hungarian human G8P[14] strain, BP1602/04, which was wholly of artiodactyl origin [49]. In this review we designate this genotype/lineage constellation as constellation pre-A. Consequently, these constellation pre-A strains contained one more animal-derived gene than the earliest Vietnamese strain (RVN1149 in Figure 4). Thus, there were a total of five bovine (artiodactyl)-derived genes, including the G8 VP7 gene. Interestingly, all G8P[8] strains possessing constellation pre-A ceased to circulate in the following year (2014), being replaced by constellation A strains (SKT-281 in Figure 4) [45].

Second, the bovine-like G8P[8] strains such as 5CR11 that was detected in Thailand from 2018–2020 had constellation E [46]. Additionally, the same constellation E was also carried by Chinese G8P[8] strains collected during the 2021/2022 winter season [47] from which all artiodactyl-derived backbone genes had been eliminated (GZ-0005 in Figure 4). We interpret that these later Thai and Chinese G8 strains as well as many Vietnamese constellation E strains were descendants of the early Thai G8 strain possessing constellation pre-A.

The lesson learned here is that a long-term continuous surveillance is necessary to provide a legitimate interpretation of discrete, snapshot observations.

#### 6.2.3. Estimated Time of Jumping from an Artiodactyla to Humans

**Figure 5:**
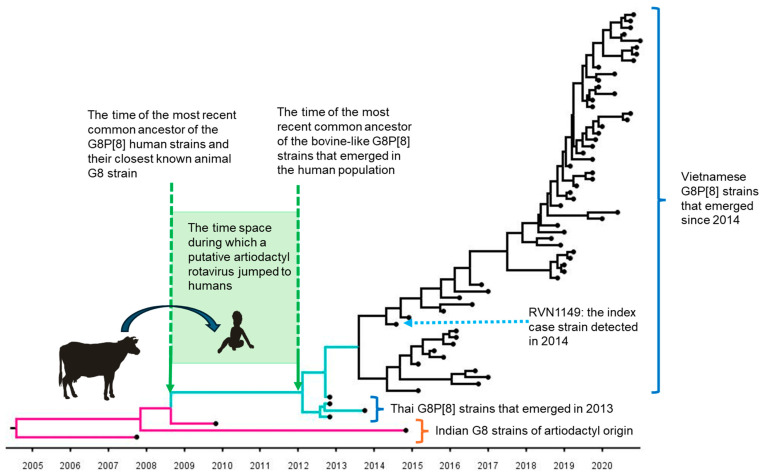
Time-scaled phylogenetic tree for the VP7 gene of bovine-like G8P[8] strains (Vietnam and Thailand). A schematic drawing of the time-scaled phylogenetic tree for the VP7 gene of bovine-like G8P[8] strains detected in Vietnam and Thailand is shown, which estimates the time of emergence and subsequent spread in the human population. The interspecies transmission of the artiodactyl G8 rotavirus strain is interpreted to have occurred sometime between 2009 and 2012. The position of the index case strain, RVN1149, which emerged in Vietnam in 2014, is indicated in the tree. The tree topology shows that RVN1149 is derived from a Thai cluster of bovine-like G8P[8] strains that emerged in 2013. Adapted from reference [44] which was published under the BB-CY license.

A time-scaled phylogenetic analysis (using a Maximum Clade Credibility tree) of the VP7 genes estimated that the initial interspecies transmission of the presumptive bovine G8 rotavirus to humans occurred between 2008 and 2012 [44] (Figure 5). Independently, Degiuseppe et al. [50], using a distinct G8 VP7 sequence dataset, estimated that the globally circulating G8P[8] strains (VP7 lineage IV), which surged to an 18% detection rate in Argentina in 2018 [51], likely originated in Thailand around 2010. This independent conclusion concurs with our own. These converging cross-species time estimates affirm our hypothesis that we observed the adaptive process 2–6 years after the putative, ancestral bovine strain crossed the host boundary.

In conclusion, evolutionary changes occur in the backbone genes of an animal rotavirus following its transmission to a new host species. Specifically, the animal-derived genes appear to be progressively replaced by cognate human-derived genes through genetic reassortment in the process of adaptation to the human host. This observation highlights a key mechanism of host adaptation in newly emerged strains.

## 7. Do G8 Strains Endemic in Africa Reflect Repeated Introductions of Bovine-to-Human Interspecies Transmission?

### 7.1. Unexpectedly High Prevalence of G8 in Africa

Prior to the emergence of bovine-like G8P[8] strains in Southeast Asia, G8 was an uncommon genotype in human rotaviruses, accounting for less than 1% of the strains detected globally [52,53,54]. However, more than 10% of strains were typed as G8 in Africa, including Malawi [55,56]. To examine the relationship to ordinary human rotavirus strains, we analysed Malawian G8 strains at the whole genome level by RNA-RNA hybridisation [34]. We concluded that Malawian G8 strains were most likely single VP7 gene substitution reassortants between bovine rotavirus (providing the G8 VP7 gene) and DS-1-like human rotavirus (providing the remaining 10 genes). As there would be many opportunities for animal–human contact to occur in rural Africa, including cattle and buffalo, it was inferred that such interspecies transmission would frequently occur [34]. Consequently, a high frequency of bovine rotavirus introductions was speculated to maintain the observed high G8 prevalence. The current understanding of this mechanism is discussed in the next section.

### 7.2. Evolution of Malawi G8 During 10-Year Surveillance

**Figure 6:**
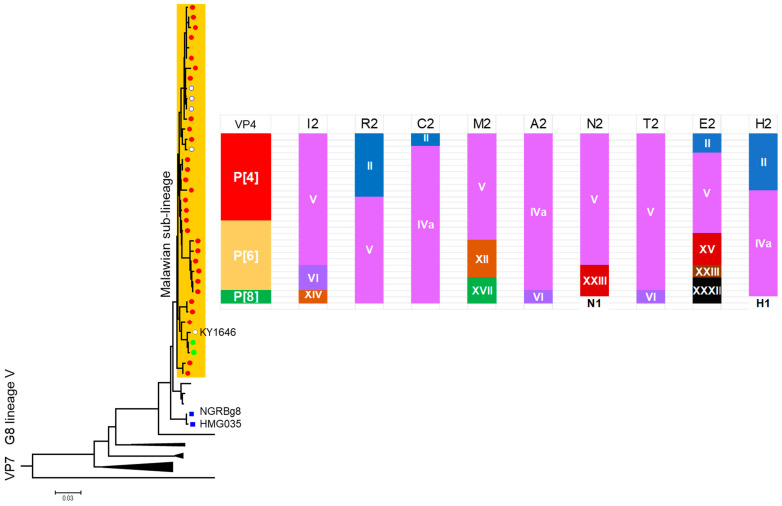
Phylogenetic structure and genomic diversity of G8 rotavirus strains in Malawi (1997–2007). This schematic drawing illustrates the phylogenetic structure of the 27 G8 strains detected in Malawi over ten years (1997–2007). The VP7 genes of these Malawian strains form a monophyletic sub-lineage within lineage V of the G8 VP7 tree. The VP4 genes consist of P[4], P[6], and P[8], with the relative frequency of each genotype proportional to the area it occupies. The segments from I2 (left) to H2 (extreme right) represent the nine internal virion protein and non-structural protein genes that form the genomic backbone of the Malawian G8 strains. Each of these nine backbone segments contains two to six different lineages, the relative frequency of which is also proportional to the area occupied by each lineage. This broad array of sub-genotype phylogeny contrasts sharply with the limited divergence and monophyly observed in the VP7 gene. This contrast suggests the frequent occurrence of reassortment among co-circulating strains that share the DS-1-like backbone genes. A closed red circle (●) represents one of the 27 G8 strains detected in Malawi. The open circle (○) labelled KY1648 represents the VP7 gene of KY1648, a simian strain from Kenya experimentally infected with a human G8P[8] strain, and the open circles with no label show G8 strains from other locations in Malawi and South Africa. Two closed green circles represent G8 strains from Democratic Republic of Congo. The two blue squares (■) represent two G8P[1] strains from human (HMG035) and bovine (NGRBg8) samples from Nigeria. This schematic figure was created anew, using the data published in ref. [57] with lineage designation following ref. [40].

Ten years after our initial identification of G8 strains in Malawi, we conducted a whole-genome sequencing analysis to answer the question of whether bovine G8 virus frequently crossed the host-species boundary to infect humans, thereby maintaining the high prevalence of G8 rotavirus strains over the study years [57]. Our findings, when taken together, indicated otherwise. The Malawian G8 strains collected in a sentinel hospital, Blantyre, Malawi, during the 10-year period (1996–2007) were genetic reassortants with the DS-1-like backbone genes (I2-R2-C2-M2-A2-N2-T2-E2-H2) and a human-endemic P genotype (P[4], P[6], or P[8]). These P genotypes are classified as P genogroup [II] and have important implications in terms of host range restriction of rotaviruses [58,59]. Thus, the VP4 spike-protein genes and the backbone genes are those of typical human rotaviruses. The VP7 genes of all Malawian G8 strains formed a monophyletic cluster within lineage V with a few other strains from Kenya, the Democratic Republic of Congo, and South Africa as well as other locations in Malawi [60] (Figure 6). Within this cluster, however, there was no G8 VP7 gene of animal rotavirus origin. Furthermore, the nucleotide sequence identities of the VP7 genes of the 27 study strains were very high, ranging from 96.9% to 99.1%. Thus, we concluded that there was only a single introduction of a bovine (or some artiodactyl) rotavirus strain to humans sometime in the not-too-distant past.

### 7.3. Incidental Observations of an Experimental Human-to-Animal Transmission

Incidentally, the VP7 phylogenetic tree includes a highly homologous G8 VP7 gene from a Kenyan simian rotavirus, KY1646 (Figure 6). This virus was not a natural isolate but a result of an experimental infection of a monkey with a human G8P[8] strain [61]. This demonstrates that if human-to-simian interspecies transmission were to occur in nature, it would present the same pattern, simian sequence nested within human sequences, in the phylogenetic tree. The implications of the G1 VP7 gene identified in a porcine rotavirus will be revisited later in this review (see Section 11.2.).

### 7.4. G8 Strains That Ended in Spillover Events Without Inheritable Consequences

Adah et al. [62] reported two G8P[1] strains from human (strain HMG035) and bovine (NGRbg8) samples from Nigeria. The G8 VP7 sequences belonged to lineage V, but formed a distinct sub-lineage separable from the Malawian G8 strain cluster with up to 5% divergence (Figure 6). The pair represents a case of bovine-to-human interspecies transmission that failed in wider human-to-human spread [63]. The genotype constellation of these Nigerian strains, G8-P[1]-I2-R2-C2-M2-A11-N2-T6-E2-H3, characteristic of a typical bovine rotavirus genotype supports the hypothesis that HMG035 was a bovine rotavirus itself that jumped to a human child [63].

Similarly, a few sporadically identified human G8P[14] strains have been documented, which are indicative of possible zoonotic transmission from artiodactyls to humans; for instance, BP1062/04 was detected in Hungary in 2004 [49], strain 2009726790 detected in Guatemala [64], strain 12597 detected in Japan in 2014 [65], A75 in Kenya in 2000 [66], PR/1300/04 in Italy in 2004 [67]. Most of these strains exhibited the consensus genotype constellations characteristic of G8P[14] artiodactyl rotaviruses: G8-P[14]-R2/R5-C2-M2-A3/A11-N2-T6-E2/E12-H3 [68]. Thus, they can be regarded as instances of direct whole-virion transmission without subsequent human-to-human spread.

These G8P[1] and G8P[14] strains of artiodactyl origin represented simple spillover events, affecting only the directly infected patients and failing to contribute to the human rotavirus gene pool or its subsequent diversification of the genome.

## 8. What Factors Enable Animal Rotavirus Strains to Adapt to the Human Host?

### 8.1. The Role of the VP4 Spike Protein in the Initial Binding to the Heterologous Host Cell

An important question concerns the factors that enabled the bovine G8 strain—ancestral to African G8P[6], G8P[4], and G8P[8] rotaviruses with DS-1-like backbone genes—to successfully adapt to the human host, in contrast to the G8P[14] strains mentioned above, which did not achieve such adaptation.

To address this question, the VP4 spike protein, more specifically, its proteolytic cleavage product VP8*, is a primary factor to consider because it is responsible for determining viral tropism and host range in rotaviruses [69,70,71]. Based on the phylogeny of the VP8* subunit, Liu et al. [58] classified 71 animal and human rotavirus strains into five P genogroups, P[I] through P[V]. P[I] includes 23 animal-associated genotypes such as P[1], P[5], P[7], etc.; P[II] includes genotypes P[4], P[6], P[8], and P[19]; and P[III] includes genotypes P[9], P[14], and P[25]. By testing recombinant VP8* molecules, Liu et al. [58] revealed a critical relationship: VP8* molecules from genogroup P[II] strains bind to the human H type 1 and Lewis b antigens, whereas VP8* molecules from genogroup P[III] strains bind to the human type A antigen. This suggests that a crucial factor for a rotavirus strain to initially cross the host species barrier and infect humans is the possession of an appropriate VP8* protein capable of binding to one of these polymorphic Histo-Blood Group Antigens (HBGAs).

Therefore, the P[14] VP8* molecule (from genogroup P[III]) likely provided the G8P[14] strain with the ability to infect human cells through the binding to human type A antigen, making it one of the bovine strains capable of initial zoonotic spillover. However, since the G8P[14] strain failed to establish widespread endemic status like the African G8P[6], G8P[4], and G8P[8] strains, all of which belong to human-adapted genogroup P[II], its VP8* binding capacity alone was insufficient for the second, more critical step: establishing sustained human-to-human transmission chains.

### 8.2. The Role of the DS-1-like Backbone Genes in Sustained Transmission

Given that both the Wa-like and DS-1-like genotype constellations are prevalent in human rotaviruses, it is plausible that the ability to establish sustained human-to-human transmission chains following a zoonotic jump is dependent on the acquisition of one of these two established human rotavirus backbone gene constellations.

In this context, it is particularly noteworthy to remind us of the successful G8 strains. Both the endemic G8 strains in Malawi and the epidemic bovine-like G8P[8] strains detected in Vietnam share the DS-1-like backbone genes in addition to human-adapted genogroup P[II] VP8 gene. This commonality suggests that the DS-1-like backbone genes carry host-range determinants essential for sustained human-to-human spread. Crucially, the Vietnamese G8P[8] strains demonstrate a clear evolutionary trend of a gradual elimination of residual bovine-derived backbone genes through successive reassortment with co-circulating human strains to achieve optimal fitness in the human host [44] (Figure 4).

### 8.3. Bovine-like G8P[8] Strains That Failed to Spread Despite the DS-1-like Backbone Genes

Medeiros et al. [48] reported a bovine-like G8P[8] strain detected in Brazil in 2017 (IAL-R193) had the same genotype/lineage constellation as what we call constellation A of the Vietnamese G8P[8] strains in addition to the same lineage IV of the G8VP7 gene (Figure 4). However, IAL-R193 and other G8 strains with DS-1-like backbone genes did not spread in the country. Medeiros et al. [48] argued by comparing the bovine-like G8 strains failing to spread with equine-like G3 causing an epidemic in Brazilian population, that bovine-like G8 strains did not achieve the fitness required to become a successful human pathogen in Brazil.

In sharp contrast, Degiuseppe et al. [51] reported that lineage IV G8 strains bearing the P[8] VP4 and DS-1-like backbone genes suddenly increased their prevalence from 0% in 2017 to 18% in 2018 in Argentina, caused an epidemic spread in Argentina.

These contrasting studies show that some further factors are necessary for an emerging strain to cause an epidemic rather than merely maintaining human-to-human transmission chains. Such factors may include competition among co-circulating strains carrying other genotype constellations, genetic susceptibility related to the prevalence of HBGA genes in the local population, and herd immunity formed by universal vaccination.

## 9. Re-Evaluation of the Phylogenetic Tree Revealed the Origin of Contemporary G1 Human Rotaviruses

### 9.1. A Super-Short Strain Was Found

The study of a rotavirus strain with an unusual character may often provide a new insight. The isolation and characterization of the AU19 strain is a case in point. Below is a case detailing this.

**Figure 7:**
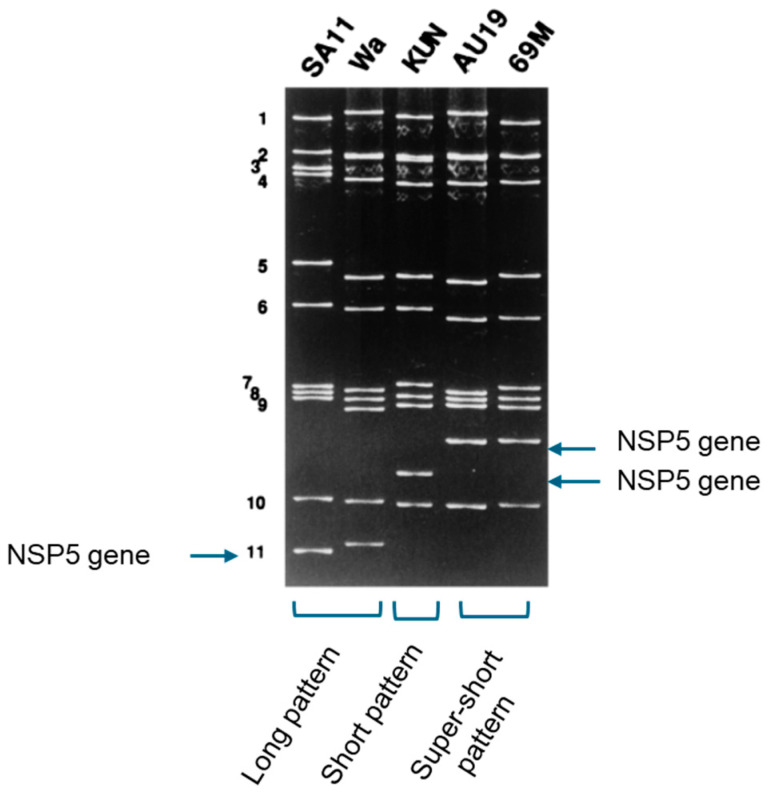
Polyacrylamide gel electrophoresis of AU19 in comparison with a few reference strains representing long, short and super-short patterns. The name of the strain is indicated above each lane. The distinct differences observed in the overall migration pattern, particularly between long-pattern strains and short/super-short-pattern strains, are primarily driven by the migration rate of genome segments 10 and 11. Although segments 10 and 11 are involved, the true molecular basis for the difference lies in the distinct migration rates of the NSP5 gene (indicated with an arrow). Reproduced from ref. [72] with permission from American Society for Microbiology.

In 1997, a 13-month-old girl, who had undergone a successful liver transplant and was under immunosuppressive therapy, was admitted to the University hospital due to severe dehydrating diarrhoea. The rapid antigen test on her stool specimen (97A019) was positive for rotavirus. As a confirmatory backup to rule out a false-positive result often encountered with immunoassays, RNA polyacrylamide gel electrophoresis was performed, revealing a “super-short” RNA pattern (Figure 7). The finding was surprising given that only a few strains with super-short RNA patterns had been reported in the world.

### 9.2. The Strain Has a New P Serotype

The reverse-transcription PCR genotyping assay showed that 97A019 contained a G1P[6] strain. Quite wrongly in retrospect, we thought at that time that there was nothing interesting in its G1 character. Instead, what captured our attention was its P[6] genotype, as it represented the first human rotavirus with P[6] to be found in Japan. We therefore isolated this strain in MA104 cells, plaque-purified it three times before making the seed stock and designated it AU19 for subsequent studies. Additionally, it was confirmed that AU19 had an identical electropherotype with the one present in the original stool specimen 97A019. It feels like a lifetime ago that such tedious procedures were routinely performed before the strain in question was subjected to further serological characterisation. At the time, researchers took it for granted that serological characterization using neutralization assays was essential for exploring biological significance of a strain.

The neutralization test of AU19 yielded a clear result for its G serotype, confirming it as G1 with conventional hyperimmune sera as well as a G1-specific monoclonal antibody. In contrast, while the P serotype was confirmed by neutralisation assay with a P2-specific monoclonal antibody, neutralisation assay with conventional hyperimmune sera indicated a significant deviation from the known P2 serotype, leading to the conclusion that AU19 was a new subtype of P2; i.e., P2C [72]. To our knowledge then, this subtype was distinct from any human or porcine rotavirus strain, making it impossible to determine its host species origin. In the meantime, we sequenced the VP7 gene of AU19, hoping to gain any hint to its host species origin, only to find that its VP7 gene formed a lineage distinct from any human G1 rotaviruses or porcine strains that were available in the DNA databases [73].

### 9.3. Analysis by Whole Genome Sequencing

Fifteen years later from the isolation of AU19, we sequenced the whole genome of AU19 and found that it had a genotype constellation of G1-P[6]-I5-R1-C1-M1-A8-N1-T1-E1-H2, a pattern typical of porcine rotavirus, except for the NSP5 gene, i.e., H2b. [74]. The H2b gene was so rare and only found in human super-short RNA strains such as 69M [75] and B37 [76] both of which were isolated in Indonesia. This finding made it improbable that the parent of AU19 reassorted with a very rare human rotavirus strain at or around the time of its transmission to humans. So, even after the whole genotype constellation of AU19 was determined, the origin of the NSP5 gene remained puzzling.

Prompted by the hypothesis that the original host species of AU19 was a pig, we gave a second look at its G1 VP7 gene to determine if AU19 was of porcine rotavirus origin rather than human. While abundant in human rotaviruses, G1 was rarely found in animal rotaviruses and only about 1% of porcine rotavirus strains were known to possess the G1 genotype, and they were mostly found in the 1970s–1980s [77]. In fact, recent surveillance seldom found G1 in pigs, apart from a single porcine G1 strain, P21-5, from Slovenia in 2004 [78]. Clearly, placing the AU19 G1 sequence within the porcine rotavirus G1 VP7 phylogeny required more samples from the last quarter of the 20th century. So, we sequenced the VP7 genes of three G1 porcine rotavirus strains that we were able to obtain: two isolated in 1980 [79] and one in 2001 [80].

**Figure 8:**
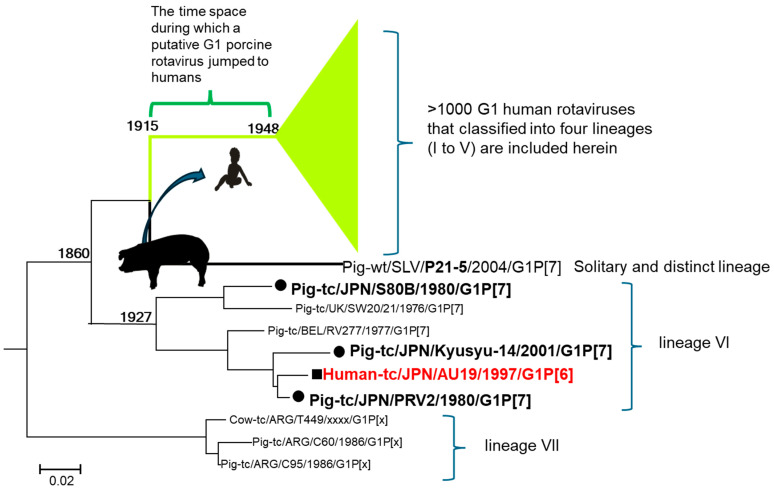
Time-scaled phylogenetic tree for the VP7 gene of G1 strains including porcine-derived human strain AU19. All endemic human G1 strains are more than 90% identical [81] and are grouped into five lineages (I to V), which are collapsed and coloured green. The putative ancestral G1 porcine rotavirus is estimated to have crossed the species barrier to humans sometime between 1915 and 1948 and became the precursor to the VP7 gene of all existing G1 human strains. The VP7 sequence of AU19 (coloured red) is nested within lineage VI and is a wholly porcine rotavirus. The strain AU19 is a case of direct transmission of a porcine rotavirus to the human child, terminating a dead-end infection. Adapted from reference [81] with the permission of the publisher.

This attempt yielded a compelling phylogenetic tree, revealing that the AU19 strain was nested within two porcine rotavirus strains isolated in Japan, PRV2 and Kyusyu-14, suggesting that its G1 gene originated from a porcine rotavirus (Figure 8) [81]. Most probably, AU19 represented a porcine rotavirus strain commonly circulating in Japan at that time. The phylogenetic tree also illustrates the remarkable diversity of the porcine rotavirus G1 gene (Figure 8). For example, the S80B strain, isolated in Japan in 1980, does not belong to the same cluster as the PRV2 strain isolated in Japan in the same year. This stands in stark contrast to the human rotavirus G1 gene, where over 1000 registered strains are concentrated into a single, tightly packed cluster with more than 90% sequence identity [81] and branched into well-established lineages I to V [82]. The most recent common ancestor of these human rotavirus G1 genes dates back to 1948 (Figure 8).

### 9.4. The Timing of the Introduction of the G1 VP7 to Humans

A closer look at the P21-5 porcine rotavirus, detected in Slovenia in 2004, shows that the time to the most recent common ancestor with all human rotaviruses is 1915 (Figure 8) [81]. This suggests that sometime between 1915 and 1948, a porcine rotavirus with the G1 gene successfully crossed the species barrier and infected humans. Through subsequent genetic reassortment, this G1 gene is believed to have become a permanent part of the human rotavirus genome, by which we mean that the ancestral G1 porcine rotavirus lost the rest of 10 genes just like the bovine-like G8P[8] rotavirus did as we discussed earlier in Section 6.2, allowing it to spread from human to human. Extrapolating from the findings, all existing human rotavirus G1 genes are considered to have originated from porcine rotaviruses. In contrast, the AU19 case represents what is known as a dead-end infection. This occurred when a porcine rotavirus successfully crossed the species barrier and infected a human, but it lacked the crucial opportunity to undergo the necessary gene reassortment and adaptive mutations that were required to establish human-to-human transmission chains.

### 9.5. The Puzzle Was Solved

Meanwhile Nagai et al. [83] published an interesting paper describing three porcine rotavirus strains found in Japan in 2014. These strains were noted for their H2b genotype in the NSP5 gene and a super-short RNA pattern on polyacrylamide gel electrophoresis. Nagai et al. [83] concluded that porcine rotavirus strains with the H2b genotype circulated in Japanese pigs. The new findings resolved the puzzle of how AU19—possessing an H2 gene and a super-short RNA pattern—came to be and caused an infection in a Japanese infant in 1997. It was not as we had to hypothesise in 2014 [74] but AU19 was wholly porcine rotavirus. Furthermore, much evidence to the commonality of H2b genotype of porcine rotavirus strains in Japan was published by Nagai’s group [84]. Thus, 27 years after the isolation of AU19, we concluded that the girl was infected with a G1P[6] porcine rotavirus.

## 10. Discussion

### 10.1. From Spillovers to Epidemics

While spillover events of animal rotaviruses to humans likely occur constantly, only two novel animal-rotavirus-derived strains, i.e., the bovine-like G8P[8] strains [41] and equine-like G3P[8] strains [42] have achieved global spread in the last two decades. The scarcity highlights the significant obstacles that animal rotavirus strains must overcome to successfully emerge in the human population.

Despite the global detection of the bovine-derived G8P[8] strain, the patterns of its establishment have been heterogeneous. In several countries, including Vietnam [41,44], Thailand [45,46], China [47], and Argentina [50,51], the strain demonstrated clear sustained human-to-human transmission leading to epidemics. Conversely, in other countries like Brazil [48], the strain was detected only sporadically and failed to persist or become endemic. This distinction suggests that successful emergence requires more than just successful initial spillover and subsequent geographic dispersal. The progression from sporadic detection to sustained transmission is plausibly influenced by a complex interplay of local factors, including the degree of genomic adaptation of the zoonotic strain to the human host; competition with dominant, co-circulating human rotavirus strains; local host HBGA profiles, which can mediate susceptibility and transmission; and overall population immunity against the emergent G genotypes. A successful global emergent strain must therefore not only overcome the initial species barrier but also navigate a variety of ecological and immunological barriers to achieve sustained and expanded transmission networks.

To better understand and analyse this complex sequence of evolutionary hurdles necessary for sustained emergence, Dennehy [85] dissected it as a three-step process.

1. Acquisition of the ability to infect a new host’s cells.
2. Adaptative mutations to the novel host, facilitating transmission between individuals.
3. Epidemic emergence in the host population.

**Figure 9:**
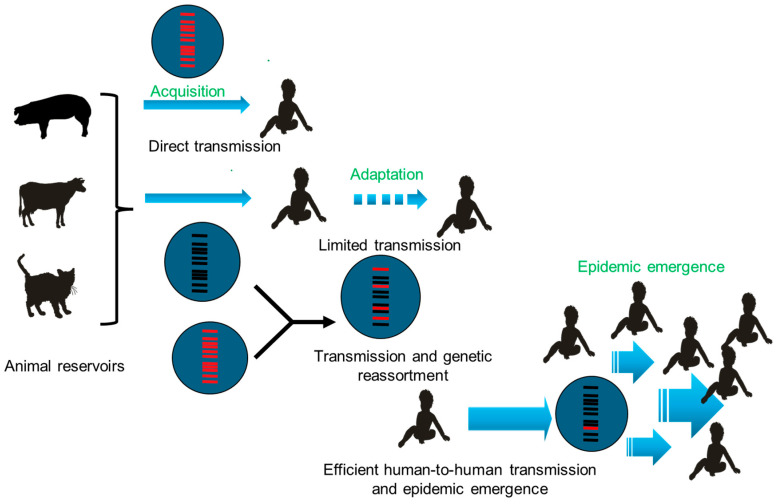
Three-step model for epidemic emergence of novel animal-derived rotavirus strains. This illustration dissects the process of animal rotavirus transitioning from spillover to epidemic emergence into three phases. Spillover (Top row): Direct animal-to-human transmission occurs after the virus acquires the ability to infect human hosts. Limited transmission (Middle row): The spilled-over strain genetically adapts to humans (e.g., via reassortment), enabling limited human-to-human transmission. Epidemic emergence (Bottom row): The adapted strain achieves competitive dominance, establishing efficient human-to-human transmission networks and causing an epidemic. Created anew for this article.

These three steps are customized for rotaviruses and illustrated in Figure 9.

#### 10.1.1. Acquisition

A major constraint on the successful cross-species acquisition of an animal rotavirus is obtaining an appropriate VP4 gene. The VP8* subunit of the VP4 protein forms the globular head of the spike and mediates the initial binding of the virion to the cellular receptor (e.g., sialic acid molecules of HBGAs) [71]. For cattle or artiodactyl strains, the possession of P[14] VP4 (genogroup P[III]), which has been shown to bind to Type A antigen, appears more advantageous for human infection than P[1] VP4 (genogroup P[I]). Although P[1] is as common as P[14] in the bovine host, it exhibits significantly lower affinity for human cells. Consequently, a P[1] rotavirus likely requires a high exposure dose to infect humans, as suggested by the clinical history of the Israeli boy. Furthermore, even if a heterologous rotavirus strain successfully infects a human, the resultant small yield of progeny viruses is often insufficient for efficient human-to-human spread.

#### 10.1.2. Adaptative Mutations

Our observation of bovine-like G8P[8] strains in Vietnam suggests that the replacement of animal-derived genes with their human counterparts via genetic reassortment is a critical factor in adaptation (Section 6.2). Here, genetic reassortment plays a crucial role by enabling the acquisition of necessary host-specific gene sets, offering a much faster evolutionary path than relying solely on the accumulation of point mutations in critical gene regions.

While the precise molecular basis enabling efficient inter-individual transmission still requires further study, some non-structural proteins such as NSP1 and NSP3 likely play a role, as detailed in Section 11.1.

#### 10.1.3. Epidemic Emergence

The final stage is the sustained human-to-human transmission of the novel animal-derived strain and its competitive dominance over existing, co-circulating human rotavirus strains, leading to a fully emerging epidemic. However, answering the question of how a strain achieves competitive dominance is difficult because it is not solely determined by the virus strain itself.

To introduce nuance to the idea that a successful epidemic is dictated solely by a shared genetic backbone, we present a few examples from our own observations in Vietnam. In 2016, during the dominance of emerging bovine-like G8P[8] strains (the first wave in Figure 4), two feline-like G3 strains emerged locally, but independently. These G3 strains were almost completely (99.2–100%) identical to the whole genotype constellation of the dominant bovine-like G8P[8] strains (except for the VP7 gene), but they failed to spread widely [86]. Subsequently, from 2016 to 2017, when the bovine-like G8P[8] strains formed a trough in prevalence (Figure 4), Wa-like G9P[8] strains became the most prevalent, peaking in 2017. During this period, three DS-1-like G9P[8] strains emerged. These three strains shared the same G9 VP7 gene as the predominant Wa-like G9P[8] strains. They had distinct DS-1-like backbone genes, formed through independent reassortment events involving globally circulating DS-1-like G1P[8], equine-like G3P[8], and bovine-like G8P[8] strains [87]. These DS-1-like backbone genes should in theory qualify them to spread widely in the human population; nevertheless, none of them did.

## 11. Future Directions

Several key areas that warrant further investigation are highlighted.

### 11.1. Molecular Mechanisms Enabling Successful Spread in Humans

Further studies on the detailed interaction between the VP8* molecule and HBGAs are imperative. While the VP8* subunit’s ability to bind HBGAs is necessary for initial human cell infection, the precise molecular mechanisms that enable certain animal-derived VP4 genes to successfully bridge the initial host barrier need to be fully elucidated. This includes identifying specific amino acid residues that dictate HBGA specificity for diverse animal rotavirus strains.

Among non-structural proteins that are likely facilitate interspecies transmission include NSP1 and NSP3. Given that NSP1 is an interferon antagonist via degrading interferon regulatory factor 3 and that NSP3 is involved in shutting off the translation of host cell mRNAs [3], it is highly likely that these proteins help the virus to evade innate immune response. For example, unlike homologous strains which efficiently suppress interferon production, heterologous strains fail to do this, leading to robust interferon restriction and poor replication in human cells [88]. Thus, acquiring homologous (human rotavirus) NSP1 is crucial to replicate efficiently in the human host, enabling sustained human-to-human transmission. In this regard, it is noteworthy that all globally spreading, bovine-like G8P[8] strains had human-adapted NSP1 and NSP3 genes, even those detected earliest in Thailand that possessed constellation Pre-A (Figure 4).

### 11.2. Integrated Genomic Surveillance at the Animal-Human Interface

Enhanced rotavirus surveillance is imperative in regions characterised by frequent animal–human interface zones, particularly in Southeast Asia and Africa, where intensive contact with livestock and wildlife elevates the risk of zoonotic transmission. A central objective of such surveillance is the isolation and complete genome sequencing of rotavirus strains exhibiting a transitional genomic constellation—strains captured during the process of interspecies adaptation, prior to the complete replacement of animal-derived genes by human counterparts. Undertaking these molecular studies, however, presents considerable logistical and methodological challenges.

A recent example is the co-surveillance effort by Bwogi et al. [89], who analysed rotaviruses in humans and domestic animals in Central Uganda. Their findings documented extensive genotype diversity among animal samples, with some genotypes overlapping between species. However, their molecular analyses provided no direct evidence of interspecies transmission. The authors concluded that, despite shared ecological environments and occasional genotype similarities, rotavirus strains in humans and animals appeared to be evolving independently.

Conversely, in a study conducted in Vietnam, what is interesting is a mixed infection case of G4P[8] and G1P[8] in a pig [90] where the VP7 gene of a strain, RVA/Pig-wt/VNM/12070_4/VP7_c2, belongs to lineage II in the G1 phylogenetic tree (within the green triangle in Figure 8). This finding, reminiscent of experimental human-to-simian transmission of G8P[8] rotavirus in Kenya [61], strongly suggests an anthroponotic infection (reverse zoonotic infection)—where the pig was infected with a human G1P[8] rotavirus strain.

These results underscore the critical need for sustained, integrated surveillance—not merely to detect zoonotic spillover events but also to rigorously confirm their absence. Such dual-purpose monitoring is essential for refining our understanding of viral ecology and the evolutionary dynamics that govern the animal–human interface.

### 11.3. The Role of Rotavirus Genome Diversification in Vaccine Selective Pressure

Genomic diversification is critically important as it generates a broad array of viral variants upon which selective pressures can act. Among these pressures, vaccine-induced immunity represents the most medically relevant force shaping rotavirus evolution. Although Velazquez et al. [91] reported that global strain diversity does not significantly compromise the broad efficacy of current rotavirus vaccines, they nonetheless underscored the essential role of ongoing surveillance to monitor potential shifts in viral populations.

### 11.4. Accelerated Viral Evolution Following Interspecies Transmission

Following the breach of a species barrier, viruses often encounter novel host constraints that exert significant selective pressure on the genome. A key consequence of this host shift can be a substantial increase in the evolutionary rate of functionally critical genes. Our study of a G6P[6] rotavirus strain isolated in Ghana [92] provides a telling example. When we studied an unusual human strain that featured a G6 VP7 gene and the DS-1-like backbone genes, the G6 VP7 genes from human strains exhibited a nearly five-fold faster evolutionary rate than the rate in bovine G6 strains. This discrepancy suggests an accelerated adaptive process in the new host environment.

This finding aligns with observations across other viral families, such as the accelerated evolution of SARS-CoV-2 following spillover from humans to farmed minks [93]. In that instance, the virus evolved up to 13 times faster in mink populations, a rate driven by host-specific selective pressures.

Collectively, these examples support the hypothesis that interspecies transmission acts as a catalyst for rapid genetic change, which is essential for facilitating the emergence and sustained transmission of novel strains in a new host.

### 11.5. Virulence of Animal-Derived Rotaviruses

While we have not yet discussed how interspecies transmission might lead to increased virulence or altered pathogenicity in rotaviruses, this potential threat cannot be dismissed. Nevertheless, to the best of our knowledge, no documented evidence currently exists showing that spillover rotavirus strains cause more severe disease in humans than ordinary human strains.

Quantitatively measuring virulence requires validated tools like the Vesikari severity score (originally developed for assessing vaccine efficacy) [94] or a similar scoring system. Applying such a methodology to novel strains presents significant challenges, as robust case–control studies require a sufficient number of subjects, which are often difficult to quickly assemble for newly emerging genotypes. We did, however, conduct one relevant study [95]. Although the focus was not on animal-derived strains, we compared the modified Vesikari scores of patients infected with novel G1P[8] double-gene reassortant strains carrying the DS-1-like backbone genes against those infected with common G1P[8] strains. Our finding was that these novel reassortant strains did not cause more severe disease.

### 11.6. Definition of Animal-Derived Genes

An animal-derived gene is fundamentally a gene that originated in an animal species. While it is clear that genes from a bovine rotavirus that recently crossed into a child are animal rotavirus genes, and those consistently found in children are human rotavirus genes, the line becomes blurred for genes with a longer history of circulation in humans. Consider the G8 VP7 gene found in the bovine-like G8P[8] rotavirus strains now circulating globally. Though its ancestry is clearly bovine—making it a gene of bovine origin—it may no longer be appropriate to call it a *bovine* rotavirus gene. This raises the question of whether, through sustained replication and adaptation within the human host population, this gene has essentially become a human gene or a humanised gene. Determining the precise moment an animal rotavirus gene transitions into a human rotavirus gene—the point at which adaptation supersedes origin—is a significant conceptual challenge in molecular phylogeny.

This distinction between an animal-derived gene and a human (or humanised) gene is not merely a theoretical exercise but a frequent issue encountered in the rotavirus literature. We can cite several practical examples that highlight this ambiguity, two of which stem from our own work. First, concerning the Malawian G8 strains that circulated for over a decade, originating from a presumed single introduction of a bovine rotavirus, we ultimately designated their VP7 gene as a human G8 VP7 gene [57]. Our intent was to emphasise that the gene was not subject to repeated, ongoing introductions from the bovine reservoir. Second, in Section 11.4, we described a Ghanaian G6P[6] strain as featuring a human-derived G6 VP7 gene. This meant the gene’s immediate ancestor was not a bovine rotavirus but rather one of the novel G6 human strains isolated sporadically worldwide. A third example is found in the literature describing G8P[8] strains collected in China during the 2021/2022 winter season. The author claimed the G8 VP7 gene was of human, not bovine, origin [47]. We interpret this to mean that these Chinese G8 strains are descendants of the early Thai G8 strain, which has likely been circulating in the human population for over a decade, thus ceasing to be an animal-derived gene. In fact, prolonged human circulation has led to the accumulation of point mutations in the G8 VP7 gene, resulting in significant antigenic changes, as detailed by Chan-It et al. [46] and Hoa-Tran et al. [44].

### 11.7. The Critical Role of Mixed Infection and Deep Sequencing

The rotavirus literature, including this review, consistently highlights the critical role of genetic reassortment in driving genome diversification. Reassortment, however, requires a mixed infection, where a single cell is infected by more than one rotavirus strain. The occurrence of mixed infection has been recognized since the early days of rotavirus research, often simply noted as the presence of more than 11 genomic RNA bands on a polyacrylamide gel and generally treated as an analytical nuisance. This same recognition persists in the current era of nucleotide sequencing, but advancements in sequencing technology are beginning to change the landscape. Specifically, accurate and unbiased analysis of mixed infection cases can now yield previously inaccessible, vital information. A prime example is a whole-genome deep sequencing study of human and porcine stool samples from the same geographical region in Vietnam [90]. Among its findings, the detection of G1 and G4 strains within a single pig stool sample is particularly noteworthy. As discussed earlier (see Section 11.2), the G1 strain originated from humans. This finding identifies a precise location and time where interspecies reassortment was actively in the process of occurring, underscoring the potential for further analytical development to provide more precise and timely insights into the generation of novel strains.

## 12. Concluding Remarks and Outlook

This review explored the topic of interspecies rotavirus transmission, revisiting key studies that have shaped our current understanding. A central focus was placed on evaluating the utility and limitations of the methods used to accurately distinguish a heterologous (animal-derived) rotavirus strain from the array of homologous (human) strains.

The literature consistently highlights the critical role of gene reassortment in segmented RNA viruses [96]. This mechanism is very efficient for the incoming virus to acquire the necessary genetic diversity in its segmented RNA genome, providing a broad array of variants upon which selective pressures in the new human environment can act. The subsequent adaptation process by accumulation of favourable point mutations grants the virus the necessary fitness to establish sustained human-to-human transmission networks.

Despite these advancements, this review revealed notable ambiguities and conflicting evidence regarding the precise evolutionary mechanisms that govern successful interspecies transmission and adaptative mutations. These challenging observations identify areas where further studies are required. It is anticipated that these complex, molecular-level evolutionary questions will be most effectively resolved by harnessing the precision and manipulative power of emerging reverse genetics technology in rotavirus research [97,98].

## Data Availability

The present work constitutes a review and synthesis of previously published literature. Consequently, all data supporting the figures and conclusions drawn in this article are available in the cited publications. No primary data were created during the research and writing of this review.

## Supplementary Materials

The following supplementary information can be downloaded at https://www.mdpi.com/article/10.3390/pathogens14121230/s1. Supplementary Table S1: General structural and functional regions of the rotavirus genome that are potentially prone to adaptive evolution.

## Abbreviations

The following abbreviations are used in this manuscript:

VP	Viral protein
NSP	Non-structural protein
PAGE	Polyacrylamide gel electrophoresis
HBGA	Histo-Blood Group Antigen
