# Supplementary material for: Interspecies Transmission of Animal Rotaviruses to Humans: Reassortment-Driven Adaptation
Source: Pathogens. 2025 Dec 2;14(12):1230. doi: 10.3390/pathogens14121230 (PMC12735868; doi:10.3390/pathogens14121230)
Supplement: Supplementary file 1 [file pathogens-14-01230-s001.zip › pathogens-3989296-supplementary.pdf]

**Supplementary Table S1 General structural and functional regions of the rotavirus genome that are potentially prone to adaptive evolution**

| Segment /Protein | Main Function | Functional Regions /Domains of Interest | Relevant Mutation Sites or Areas | Biological Effect / Interpretation |
|---|---|---|---|---|
| VP7 (G) | Outer capsid glycoprotein | Antigenic regions A, C and F | Substitutions in epitopes A (aa 87-101), C (aa 208-221), F (aa 233-242) | Alter antigenicity and may enable immune escape |
| VP4 (P) | Spike protein for viral entry | Subunits VP8* (receptor binding) and VP5* (penetration) | VP8*:(aa 1-231) HBGA binding site; hypervariable region (aa 74-203); Cleavage site: 231, 237, VP5*:(aa 238-750/751) | Determines host tropism and receptor affinity |
| VP6 (I) | Middle-layer protein; major antigen | Interacts with NSP4 | Polymorphisms affecting antigenic type | Determines subgroup specificity and diagnostic typing |
| VP1 (R) | RNA-dependent RNA Polymerase (RdRp) | Catalytic domains; Nucleotide binding sites | Substitutions in the GDD motif or polymerase active site | Affects viral replication efficiency and fidelity |
| VP2 (C) | Inner most layer | RNA binding domain; Structural support | Mutations affecting capsid stability or VP1 interaction | Affects assembly and genomic RNA packaging |
| VP3 (M) | Capping enzyme; Guanylyltransferase | Methyltransferase and Guanylyltransferase domains | Sites affecting RNA capping activity | Crucial for mRNA stability and translation |
| NSP1 (A) | Interferon antagonist | IRF3-binding domain | Length variation (e.g., deletions) or substitutions in IRF3 binding domain | Modulates host innate immune response |
| NSP2 (N) | Viroplasm scaffold RNA-binding NTPase | NTPase domain; Viroplasm formation | Sites affecting RNA binding or oligomerization | Essential for viroplasm formation and genome replication |
| NSP3 (T) | Host translation shut-off | eIF4G binding site; RNA binding domain | Substitutions in eIF4G binding region | Evicts the host poly- A binding protein from its binding site in eIF4G, thereby ensuring viral mRNA molecules are preferentially translated |
| NSP4 (E) | Enterotoxin; ER localization | ● Intracelluar viral receptor ● Enterotoxin ) | Substitutions in ● VP6-binding domain (aa 161-175) ● Enterotoxin domain (aa 114–135) | ● Functions as an intracellular viral receptor captuirng nascent double-layered particles ● Modifies pathogenicity and induces diarrhea |
| NSP5 (H) | Viroplasm scaffold | Phosphorylation sites (serine/threonine); | Sites affecting phosphorylation or interaction with NSP2 | Essential for viroplasm function and genome replication |
